# Editorial: Women in neurotoxicology: 2021

**DOI:** 10.3389/ftox.2023.1248748

**Published:** 2023-09-04

**Authors:** Monica Renee Langley, Alice Villalobos, Sarah Vecchio

**Affiliations:** ^1^ Department of Molecular Pharmacology and Experimental Therapeutics, Mayo Clinic, Rochester, MN, United States; ^2^ Department of Physical Medicine and Rehabilitation, Mayo Clinic, Rochester, MN, United States; ^3^ Department of Medical Education, Texas Tech Health Sciences Center-School of Medicine, Lubbock, TX, United States; ^4^ Addiction Centre, Biella, Italy; ^5^ Italian Society of Toxicology (SITOX), Milan, Italy; ^6^Ser.D Biella-Drug Addiction Service, Biella, Italy

**Keywords:** women in STEM, neurotoxicity, neurotoxicology, women in science, women in neuroscience

Women’s participation in neurotoxicity research is fundamentally important to provide diverse perspectives during study design, execution, analysis, and interpretation of research. This includes a myriad of considerations from sex-specific vulnerabilities, environmental exposures, reproductive and developmental considerations, and underrepresentation in clinical trials, among others. Despite this, less the 30% of researchers worldwide identify as women. This Research Topic was inspired to highlight accomplished women researchers that wished to contribute their first or last author publications across the field of Neurotoxicology. By highlighting women in neurotoxicology, we also provide an opportunity for female researchers to serve as role models for the next-generation.

First, the manuscript by Carolina Caroso dos Santo Durão et al. examined exposure to environmental tobacco smoke (ETS) during the embryonic stage to see how it affected neuroinflammation in the adult mouse later in life. Multiple sclerosis (MS) is a neurological disorder involving inflammation and demyelination of the central nervous system and that has higher prevalence in women. A common mouse model of MS, called experimental autoimmune encephalomyelitis (EAE), was used in this study. In cell culture and EAE models, ETS exposure resulted in increased neuroinflammatory markers when compared to compressed air, including increased levels of proinflammatory cytokines IL6 and TNFα. Functionally, the ETS exposure resulted in significant worsening of EAE mouse clinical scores.

Next, a paper by Yaghoobi et al. investigated the developmental neurotoxicity potential of specific polychlorinated biphenyls (PCBs) in a zebrafish model. The researchers confirmed different PCB congeners exhibited varying potency in sensitizing ryanodine receptors (RYR) in zebrafish muscle. They found that a subset of these PCB congeners altered photomotor behavior in larval zebrafish, and the pattern of behavioral effects corresponded to the pattern of RYR sensitization, providing *in vivo* evidence supporting the hypothesis that RYR sensitization contributes to the developmental neurotoxicity of PCBs.

A review paper authored by Albrecht et al. summarizes effects of developmental lead (Pb) exposure on ethanol (EtOH) responses in *Caenorhabditis elegans* (*C. elegans*), a powerful model organism used to elucidate toxicant mechanisms. The authors describe morphological changes in dopamine synapses and dopamine-dependent behaviors, providing insights into the neurobiological mechanisms underlying the relationship between these neurotoxicants, and highlights the utility of *C. elegans* as a model for studying combined neurotoxicant effects.

Finally, a rat study by Reyes-Bravo et al. explored the role of chronic exposure to the herbicide atrazine to assess GABAergic and glutamatergic systems. After 1 year, there were changes in vertical activity episodes and several genes within the glutamatergic and GABAergic systems in the brain regions explored, which included striatum, nucleus accumbens, ventral midbrain, prefrontal cortex, and hippocampus. Of these, striatum had the most changes, followed by hippocampus. The results in this article spark interest to perform neurochemistry and neurobehavioral analysis especially in models of neurodegenerative disorders affecting basal ganglia.

**FIGURE 1:**
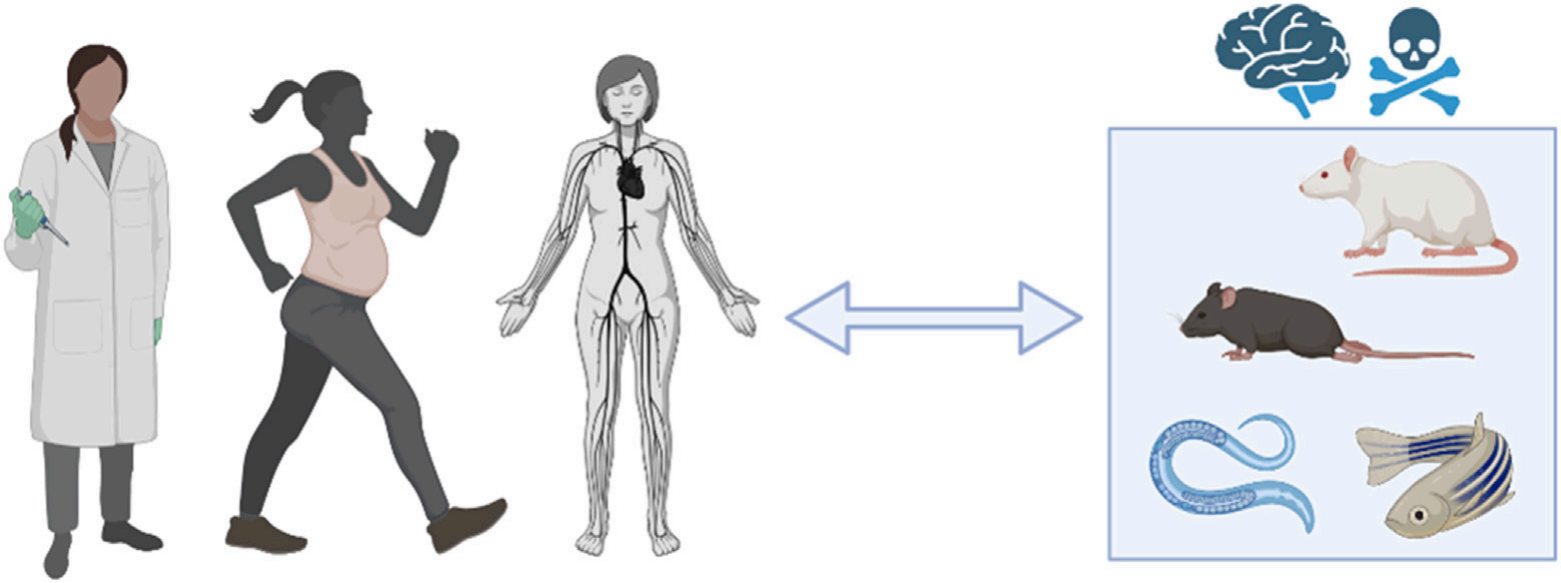
Women researchers contribute to the neurotoxicology field by providing their unique insight and using model organisms to uncover mechanisms underlying various environmental exposures relevant to risk and progression of neurodevelopmental, neuropsychiatric, neurodegenerative, and other neurological disorders affecting women and their communities. Schematic generated in Biorender.

Overall, the research studies uncover associations between environmental exposures and neurodevelopmental outcomes across several model systems and stages of development. The impact of environmental factors on neurological disease development has implications for the health of women across their lifespan in addition to their families, but also provides research communities valuable insight into the direction of future scientific endeavors Figure 1.

